# A Detailed dSPACE-Based Implementation of Modulated Model Predictive Control for AC Microgrids

**DOI:** 10.3390/s23146288

**Published:** 2023-07-11

**Authors:** Ariel Villalón, Carlos Muñoz, Javier Muñoz, Marco Rivera

**Affiliations:** 1 Engineering Systems Doctoral Program, Faculty of Engineering, University of Talca, Campus Curicó, Curico 3344158, Chile; 2 Department of Electrical Engineering, Faculty of Engineering, University of Talca, Campus Curicó, Curico 3344158, Chile; carlosmunoz@utalca.cl; 3 Estudiante de Doctorado, Departamento de Ingeniería Eléctrica, University of Jaén, Campus Lagunillas s/n, Building A3, 23071 Jaén, Spain; 4 Laboratory of Energy Conversion and Power Electronics, Faculty of Engineering, University of Talca, Campus Curicó, Curico 3344158, Chile; marcoriv@utalca.cl; 5 Power Electronics and Machine Centre, Faculty of Engineering, University of Nottingham, Nottingham NG7 2RD, UK

**Keywords:** AC microgrid, droop control, dSPACE, fixed-switching-frequency modulated model predictive control, power sharing, voltage source inverter

## Abstract

Microgrids represent a promising energy technology, because of the inclusion in them of clean and smart energy technologies. They also represent research challenges, including controllability, stability, and implementation. This article presents a dSPACE-control-platform-based implementation of a fixed-switching-frequency modulated model predictive control (M${}^{2}$PC) strategy, as an inner controller of a two-level, three-phase voltage source inverter (VSI) working in an islanded AC microgrid. The developed controller is hierarchical, as it includes a primary controller to share the load equally with the other power converter with its own local modulated predictive-based controller. All details of the implementation are given for establishing the dSPACE-based implementation of the control on a dSPACE ds1103 control platform, using MATLAB/Simulink for the controller design, I/O implementation and configuration with the embedded dSPACE’s real-time interface in Simulink, and then using the ControlDesk software for monitoring and testing of the real plant. The latter consists of the VSI operating with LCL filters, and sharing an RL load with a paralleled VSI with exactly the same controller. Finally, the obtained experimental waveforms are shown, with our respective conclusions representing this work, which is a very valuable tool for helping microgrid researchers implement dSPACE-based real-time simulations.

## 1. Introduction

Around the world, there has been a marked increase in the spread of distributed energy resources (DERs) in electrical distribution grids. One of the main drivers of this increase has been the inclusion of more renewable energy that may be available, scattered across several territories, allowing these DERs to be close to the demand centres (loads). This has brought benefits, such as the improved reliability of electricity provision, reduced costs, increased safety against physical and cyber hazards, assimilation of renewable energy, and a decrease in the carbon footprint [1]. According to the International Energy Agency, as supporting technologies improve, develop, and become more cost-effective, it is expected that DERs will continue to experience the increasing pace of incorporation [2].

In addition to the expansion of DERs, with renewable energy, microgrid conceptualisation has had a very positive impact on the effective control of DERs in electrical distribution networks. These novel types of power systems allow for improved reliability and resilience, compared to individual energy sources, by embedding multiple DERs [3,4,5]. One paramount characteristic of microgrids is their ability to work in both grid-connected and islanded modes [6,7,8]. It is in islanded mode that, to ensure a stable and profitable microgrid operation, the real and reactive powers of DERs should be proportionally shared by their respective power ratings [4,7,9].

There are control algorithms associated with the equal sharing of the loads in a microgrid: Droop control can do the power sharing properly, without any external communication among the different power converters that interface with the DERs [10,11,12,13,14].

In addition to power sharing control, power converters in the microgrid have to control the DGs embedded in the system. Usually, this is carried out by a linear controller, with an inner loop voltage–current feedback control [15,16,17,18].

Microgrids have smart controllers and automated modular sections, all of which must operate without any external control. Thus, the application of model predictive control (MPC) to AC and DC microgrids eases multivariable and multi-timescale implementation when hierarchical control is applied [19,20]. Finite-set MPC (FS-MPC) has a recognised drawback: the variable switching frequency produced at the output of the power electronics converter, which affects the configuration of the necessary power filters for the connection to the grid. To eliminate the drawbacks of this inner controller, modulated model predictive control (M${}^{2}$PC) appeared, as a feasible solution for power converters in a microgrid [21]. The application of predictive control-based inner controllers for power converters working in microgrids is a research area that is still immature, but there is a very interesting prospect of analysing its performance and implementation in these microgrid environments [22].

As DERs and renewable energy, together with microgrid systems and their control strategies, acquire more relevance, more research and development resources are aimed at this field. Several laboratories have been implemented, to carry out important research on microgrid planning, operation, and control, with the inclusion of DERs based primarily on renewable energy. From a structural point of view, microgrid laboratories can be classified into four categories: real microgrid; simulation-software-based microgrid; hardware-in-the-loop (HIL)-based microgrid; and hybrid microgrid [23].

**Figure 1:**
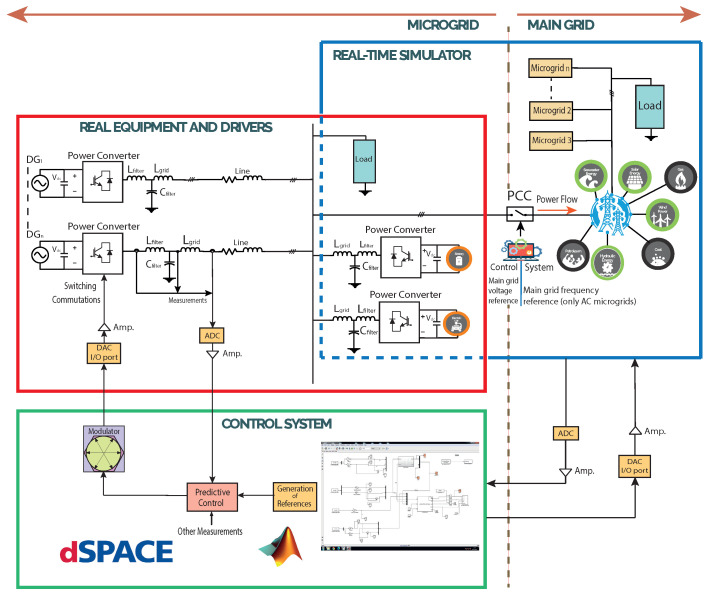
General concept of a real-time simulation dSPACE-based test setup for a typical microgrid.

In a real microgrid laboratory, the microgrid supplies local loads, and can also be used for experimental research in this field. In a microgrid laboratory based on simulation software, computers with simulation software are essential, to build the microgrid in this environment, with simulated DG, power converters that connect to these DGs, and additional but external digital or analogue modules. Nevertheless, the results of research using physical experimental systems are more reliable than simulation environments results. Usually, the concept of the hybrid microgrid laboratory, being a real plant with simulation-software-based microgrids, may fit better, for meeting reliability requirements, and achieving simple-to-implement research results. Software used in the latter case includes MATLAB/Simulink and SimPower Systems [23]. This can be seen as general concept in Figure 1, where real equipment is interacting with a simulated grid and a dSPACE-based control system.

On the other hand, the HIL-based microgrid laboratory allows for the testing of real equipment, such as DGs and distributed energy storage systems (DESS), connected at the same time to a microgrid, with its controller model that can be simulated in real time [23,24,25]. An HIL-based laboratory or testbed may allow one to distinguish the architecture that best fits a given utilisation, avoiding related costs and resources limitations, to implement real microgrid applications. Thus, the implementation of HIL-based microgrids facilitates validation of control schemes for a certain microgrid architecture [1].

A platform that is widespread in research centres and laboratories is the dSPACE control platform, which is a real-time interface (RTI) that allows for the implementation of the control system in MATLAB/Simulink with real equipment and drivers, such as power converters and loads [26,27,28]. Although the dSPACE developers have published their manual for the different models of their control platforms, there are not many publications related to implementing real-time simulation—in this case, a hybrid microgrid laboratory using these dSPACE control platforms, real systems, experimental setup of power converters, power filters, and loads.

Implementing MPC-based control is well-documented, addressing the theoretical aspects. For example, in the work developed by [21], the authors implemented an FS-MPC strategy for a three-level NPC inverter fixing the operational switching frequency. Experimental implementation of the control was carried out, using a dSPACE ds1103 control platform with a Spartan 3 FPGA connected via the I/O bus expansion that is available in the dSPACE control platform. Although the authors provided extensive and valuable details of the implementation, a detailed step-by-step implementation in the Simulink environment was not included in the manuscript. In the work of [29], an FS-MPC was implemented, to control two voltage source converters in an experimental implementation of an isolated AC microgrid. To validate the results, the author used a dSPACE MicroLabBox ds1202 control platform, without giving detailed step-by-step implementation within the control platform. When working with real-time microgrid implementations with two or more power converters, dSPACE control platforms may have limitations. Nevertheless, in the work developed by [30], a single dSPACE ds1103 control platform was used, to control two power converters: here, proportional–integral (PI) control was used as an inner controller, and to generate the three-phase PWM pulses for each converter; the authors explained the use of the Simulink blocks, which are available in the dedicated library of the software, but did not provide further details of the Simulink model to implement the controller in the dSPACE platform. In [31], the authors implemented a modified modulated MPC (M${}^{3}$PC) strategy for a grid-connected converter, using a dSPACE ds1104 platform; however, while  the authors provided the experimental parameters, they did not explain further the implementation in the dSPACE control platform.

In the specialised market of HIL control platforms, other control platforms are available, in addition to dSPACE control platforms. Their use is well-documented; however, as with dSPACE platforms, no further details of the implementation are given. For example, in a renewable-energy-based AC microgrid, the authors of [32] established an MPC strategy without any proportional–integral–differential (PID) regulator: to do so, the authors used MATLAB/Simulink and the Opal OP5700 real-time laboratory test platform (RT-LAB). Step-by-step details for the implementation were not provided. Another implementation of an M${}^{2}$PC strategy applied to LCL-filtered grid-tied inverters, using a different HIL control platform, was developed in [33]. The authors implemented hardware-in-the-loop validation, emulating the power converter, the LCL filter, and the grid using the Typhoon HIL 402 hardware-in-the-loop. Again, several valuable theoretical details were provided, but no detailed implementation using the HIL platform was included.

Beyond the application of HIL platforms to predictive control applied to microgrids, there are publications related to their application to the control of power systems and distributed generation, which provide details on the implementation of the controller and the system. For example, in the work developed by the authors in [25], the real-time simulation of a complete isolated grid was included in the simulation software environment RT-LAB of the Opal-RT platform. The construction process in MATLAB/Simulink was described, though without graphic evidence, and more details would have been useful for the reader. The implementation of a nonlinear controller using Lyapunov function for a DFIG on the dSPACE 1104 control platform was carried out by the authors in [27], in which an explanation of the dSPACE 1104 platform technical characteristics was provided, along with the inclusion of the complete control system as a Simulink model for the dSPACE 1104 real-time interface platform.

Due to the lack of well-documented and detailed step-by-step microgrid HIL implementation of M${}^{2}$PC as inner-controller of a two-level, three-phase voltage source inverter (VSI), sharing a RL load with power sharing algorithms (droop control with virtual impedance), in a dSPACE control platform, this article looks to become a valuable and useful tool for microgrid researchers interested in implementation of these kinds of controllers. The focus, as mentioned, is on the real-time implementation of microgrids using dSPACE-based experimental setups for proper and high-impact scientific results.

In this way, this article presents a dSPACE-control-platform-based implementation of an M${}^{2}$PC strategy as an inner controller of a two-level, three-phase VSI operating in an islanded AC microgrid. The developed controller works as a hierarchical controller, as it includes a primary controller for equal sharing of the load power with the other power converter, with its own local modulated-predictive-based controller. All the details of the implementation are provided, for establishing the dSPACE-based validation of the control in a dSPACE ds1103 control platform, using MATLAB/Simulink for the controller design, I/O implementation, and configuration with the embedded dSPACE’s real-time interface (RTI) in Simulink,  then using the ControlDesk software for monitoring and testing of the real plant. The VSI operates with LCL filters, and shares an RL load with another VSI with exactly the same controller. Here, it is important to mention that this work provides details of the implementation of previous published work by the same authors in [34], where comparisons with other works of simulated and experimental results were widely addressed.

The academic contribution of this paper is to provide a detailed description of the implementation of the M${}^{2}$PC strategy with the power-sharing outer loop for the dSPACE control platform. As noted, most of the previously published works refer to the theoretical aspects, but provide only a brief explanation of the implementation of the control strategies in the different available HIL platforms: dSPACE; Opal-RT; and Typhoon HIL, among others. Additionally, the contribution to the researchers of predictive control applications to power converters and microgrids, where even the inclusion of C code for the predictive controller, and the inclusion of dead-times in the FPGA, are valuable for helping building real-time implementations for dSPACE platforms.

Finally, this work, in Section 2, explains in detail all the components of the real plant, the hardware setup, where the two-level, three-phase VSI used is explained, with the LCL filters and the RL load, explaining the semiconductors used in the power converter, the development of the printed circuit board (PCB) for the LCL filter, and the necessary model of the system to develop the M${}^{2}$PC scheme. Additionally, here, the dSPACE ds1103 control platform used is explained, with an emphasis on its features, which are used to implement the controller. Then, in Section 3, the whole process of implementing the modulated model predictive controller in the real-time interface (RTI) of the dSPACE ds1103 control platform is explained, considering the controller programming, the implementation of dead-times for safe commutation of the VSI,  the inclusion of power sharing controllers of the RL load, and the testing and monitoring process of simulation in real time, to obtain proper results. The results of the experimental implementation are provided in Section 4, and are discussed in Section 5. Finally, the paper concludes with Section 6, which includes a summary Table of State-of-the-Art developments on hardware-in-the-loop and real-time simulation platforms applied to control power converters, microgrids, and distributed generation (Table 3). In addition, the code of the M${}^{2}$PC-based controller, and of the implementation of dead-times in the FPGA board, are provided in the Appendix to this paper (Appendix A).

## 2. Experimental System Description

**Figure 2:**
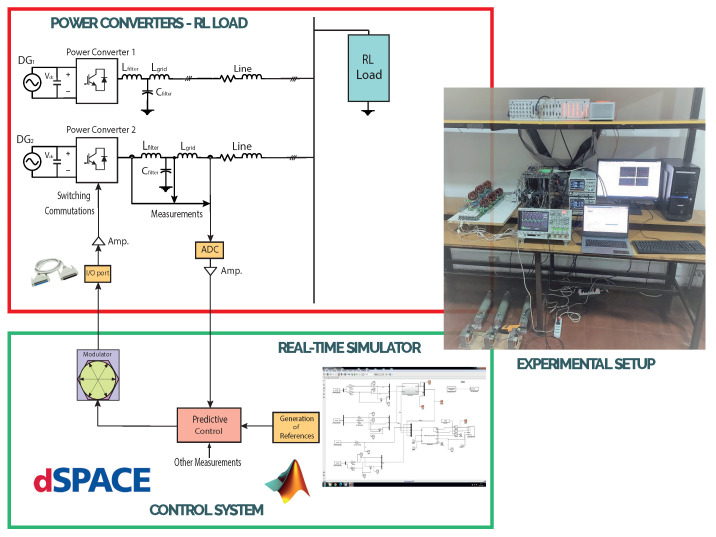
Concept of the dSPACE-based test setup for the islanded AC microgrid.

In Figure 2, the general concept of the dSPACE-based setup applied to an isolated AC microgrid is depicted: it can be seen that the microgrid is made up of two power converters, which are interfacing DGs (controllable voltage sources). In this case, each power converter had a decentralised controller that worked independently. Figure 2 shows that, specifically for this work, the inner controller for each VSI was controlled by the dSPACE ds1103 control platform. The advantages of working with the dSPACE control platform lie in its ability to work in a MATLAB/Simulink environment. All the controller programming was built in Simulink, using the dSPACE’s Simulink libraries to interface the software side with the hardware side, as can be seen in Figure 2.

### 2.1. Description of the Power Converter

**Figure 3:**
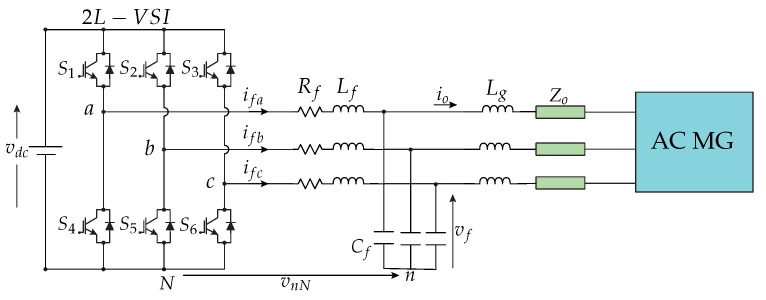
Two-level, three-phase VSI connected through an output LCL filter linked to an AC microgrid with a line impedance $Z_{o}$, as analysed in [34].

The converter implemented was a DC–AC power converter, consisting of three legs, each with two power switches, MOSFETs. In Figure 3, the two-level, three-phase VSI is shown.

**Figure 4:**
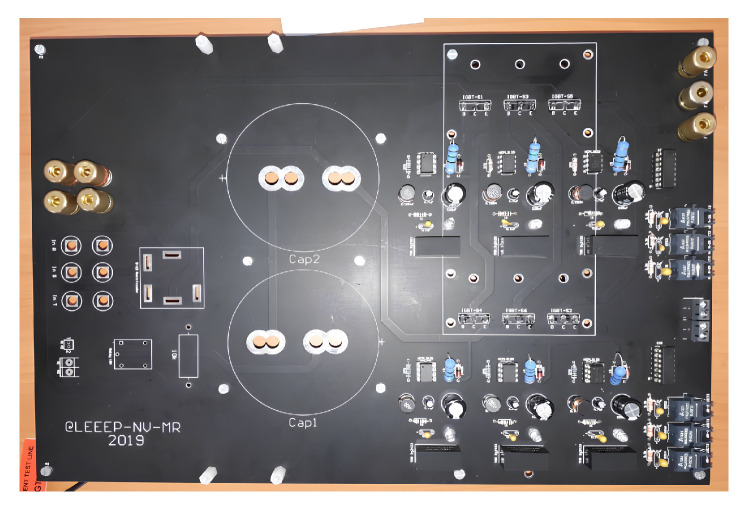
View of the implemented PCB for the two-level, three-phase VSI.

The implemented VSI was built and designed as a PCB, as can be seen in Figure 4.

VSIs are commonly found operating in AC microgrids, and, in this case, with LCL filters at the output terminals (Figure 3), to decrease switching harmonics, allowing for operation in grid-forming mode and control of the capacitor voltage of the LCL filters [29].

#### Switches Characteristics

The switches included in the implemented two-level, three-phase VSI were metal-oxide-semiconductor field-effect transistors (MOSFETs) of the model STF22N60M6, with a drain–source voltage, $V_{DS}$, value 600 V [35]. In Table 1, the electrical ratings of the MOSFETs are shown:

### 2.2. LCL Filter

The inclusion of the LCL filter was based on the need to filter the power from the voltage source to the rest of the AC microgrid. Therefore, the components of the LCL filter for each phase were determined as $L_{f}$, $C_{f}$, and $L_{g}$, which were the filter inductance, the filter capacitance, and the grid inductance, respectively.

**Figure 5:**
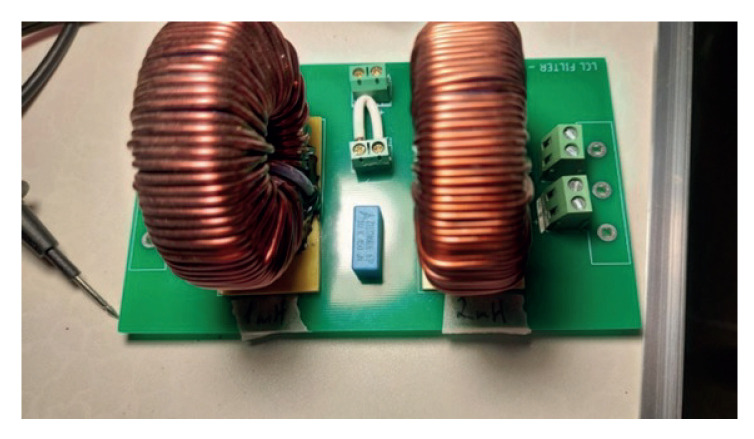
View of the experimental LCL filter.

The developed LCL filter, which exerted the coupling of the VSI with the rest of the islanded AC microgrid, is shown in Figure 5.

The size of the LCL filter was taken from a filter developed previously in the Laboratory of Renewable Energy and Electrical Conditioning (LERAE), but with a greater capacitance to improve the control of the capacitor voltage (Figure 5).

**Figure 6:**
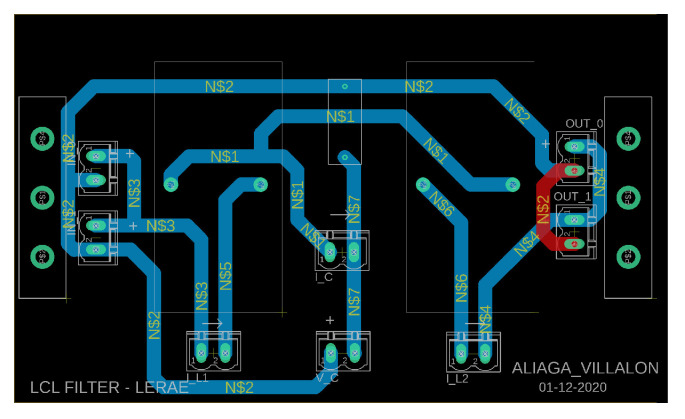
View of the PCB for the experimental LCL filter.

The LCL filter was developed using a PCB, as shown in Figure 6.

The parameters considered for the LCL filter were:

- $L_{f}$ = 2.0 mH;
- $C_{f}$ = 11 μF;
- $L_{g}$ = 1.0 mH.

In order to model the behaviour of the LCL filter, the model was developed according to the details provided in Section 2 of [34]: system description. The discrete model of the LCL filter is shown as follows:

(1) $$\begin{array}{c} x\left(k+1\right)=A_{d}x\left(k\right)+B_{d}{\vec{v}}_{inv}\left(k\right)+E_{d}{\vec{v}}_{g}\left(k\right), \end{array}$$

with (k) as the present time, (k+1) as the next sampling time, and **x** as the state vector $x={\left[\begin{array}{ccc} \vec{i_{f}} & \vec{v_{f}} & \vec{i_{g}} \end{array}\right]}^{T}$ . Taking into account that the sampling period was $T_{s}$, the discrete matrices $A_{d}$, $B_{d}$, and $E_{d}$ were explained as [33,34,36]:

(2) $$A_{d}=e^{AT_{s}},$$

and

(3) $$B_{d}=\int_{0}^{T_{s}}e^{A\tau }Bd\tau ,$$

and  

(4) $$E_{d}=\int_{0}^{T_{s}}e^{A\tau }Ed\tau ,$$

with A, B, and E as the continuous matrices of the state space model in continuous time of the LCL filter, developed in previous work published in [34].

### 2.3. RL Load

**Figure 7:**
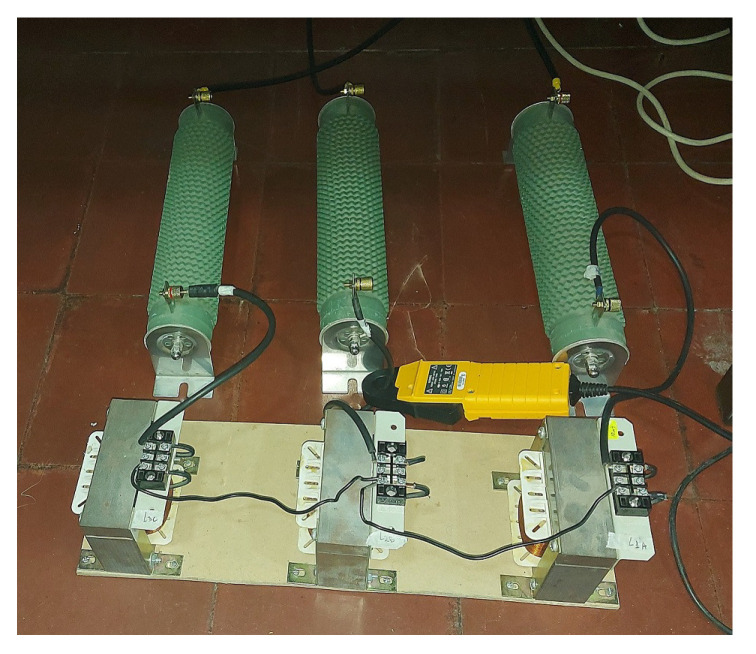
View of the experimental shared three-phase RL load.

The setup developed to establish the AC microgrid included a shared three-phase RL load, depicted in Figure 7, that constituted the rest of the AC microgrid.

The three-phase RL load had the following experimental parameters:

- $R_{l}$ = 10 Ω;
- $L_{l}$ = 10 mH.

### 2.4. dSPACE ds1103 Control Platform

The dSPACE ds1103 control platform is particularly configured to develop high-speed multivariate digital controllers and real-time simulations in several disciplines. The dSPACE ds1103 operates in complete real time for advanced applications, and considers the inclusion of a slave DSP subsystem based on the TMS320F240 DSP Texas Instruments microcontroller [37].

The specific interface connector panels of the dSPACE ds1103 platform provide easy access to all input and output signals of the dSPACE ds1103 control platform [37]:

- The CP1103 connector panel provides useful connections between the ds1103 control platform and other equipment that will be connected to it;
- In addition to the connector panel, the dSPACE ds1103 considers additional connectors and a panel with an array of LEDs showing the states of the digital signals.

The dSPACE ds1103 includes two different types of analogue-to-digital converter (ADC) for the analogue input channels [37]:

- Four ADCs with four multiplexed input signals each, from ADCH1 to ADCH16;
- Four parallel (non-multiplexed) ADCs with one input signal each, from ADCH17 to ADCH20.

In this implementation, the ADCs were used to connect multiple current clamps and differential voltage probes, to obtain readings with which to establish the control of the VSI. The ADCs that were used corresponded to those that were multiplexed.

Additionally, dSPACE ds1103, with its slave DSP, provides I/O with pins with several signals: among them, three-phase space vector PWM signals, which were used in this implementation as the modulator of the modulated model predictive controller [37].

### 2.5. Experimental Complete Setup

**Figure 8:**
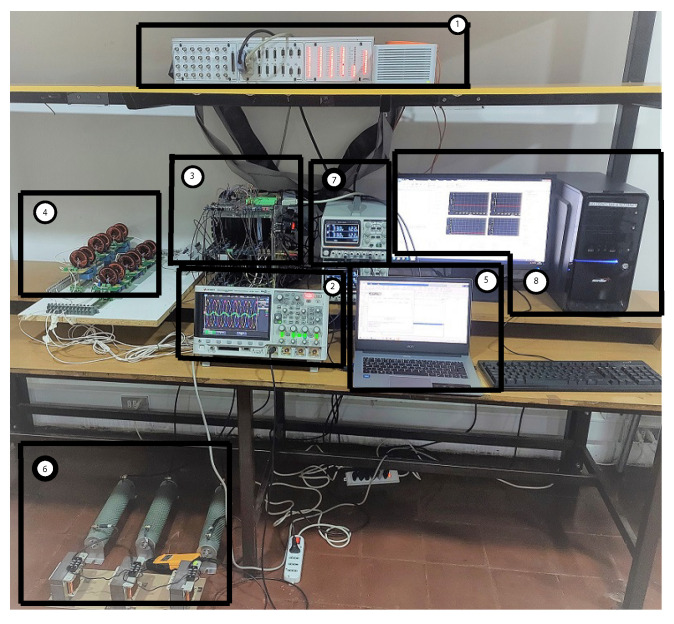
Experimental setup used in [34]: (**1**) dSPACE ds1103 control platform; (**2**) oscilloscope for signal acquisition; (**3**) two-level, three-phase VSI, Atlys FPGA, DC input; (**4**) LCL filters; (**5**) programming computer; (**6**) RL load; (**7**) controllable DC voltage source; (**8**) dSPACE platform’s programming computer.

All the components described above were put together in the laboratory, to form the experimental setup shown in Figure 8. This experimental setup was used to obtain the experimental results that verified the implementation of the M${}^{2}$PC applied to the islanded AC microgrid. In addition to all the components already described, the dSPACE ds1103 control platform can be seen in Figure 8, being the device where the controller was implemented. In addition, the FPGA was included in the experimental setup. The FPGA will be explained later in this paper.

## 3. Implementation of the Controller

### 3.1. The Modulated Model Predictive Controller

**Figure 9:**
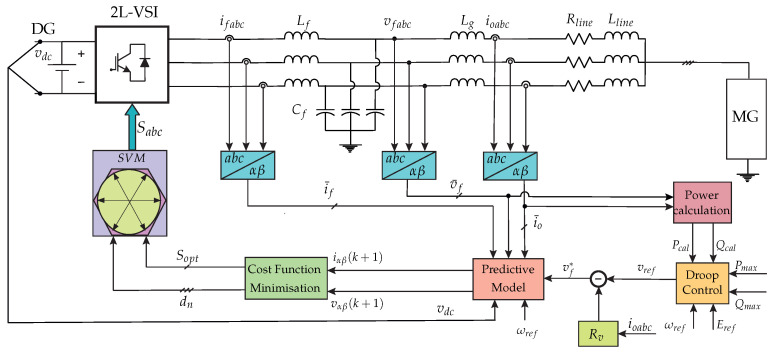
Block diagram of the modulated model predictive control scheme for a two-level, three-phase LCL-filtered VSI in islanded AC microgrid, explained in [34].

The theoretical concept of the fixed-switching-frequency M${}^{2}$PC is depicted in the block diagram of Figure 9. This control is exerted to every VSI operating in the islanded AC microgrid, and enables every VSI to be controlled independently of any other external device or communication link.

The details of this M${}^{2}$PC scheme shown in Figure 9 were widely developed and explained in the previous published work by the same authors in [34].

### 3.2. MATLAB/Simulink Model

#### 3.2.1. Modulated Model Predictive Control

The establishment of the M${}^{2}$PC algorithm is realised in the Simulink environment of the dSPACE ds1103 platform that implements the model of the LCL filter, described in detail in [34]. Then, the cost function (CF) is implemented in this algorithm, to be minimised using the predictions obtained from the mathematical model of the system. When the latter is fulfilled, the sector with its adjacent vectors obtains the duty cycles that are introduced in the space vector modulation (SVM). Then, the multi-objective CF is as follows [34]:

(5) $$g_{i}=\lambda_{i_{o}}\cdot {\left(i_{o_{\alpha \beta }}^{*}-i_{o_{\alpha \beta }}^{p}\right)}^{2}+\lambda_{v_{f}}\cdot {\left(v_{f_{\alpha \beta }}^{*}-v_{f_{\alpha \beta }}^{p}\right)}^{2},$$

where $\lambda_{i_{o}}$ and $\lambda_{v_{f}}$ are the weighting factors for the output current $i_{o}$ and the capacitor voltage $v_{f}$, respectively, $i_{o_{\alpha \beta }}^{p}$ and $v_{f_{\alpha \beta }}^{p}$ are the predicted values of the output current vector and the capacitor voltages obtained, respectively, and $i_{o_{\alpha \beta }}^{*}$ and $v_{f_{\alpha \beta }}^{*}$ are the output current reference and capacitor voltage reference, respectively.

The presence of the LCL filter poses some difficulties in the modelling of the modulated predictive controller in the isolated microgrid, where the capacitor voltage is the main control objective. The coupling of the derivative of the current in the input inductor $L_{f}$ ($\frac{di_{c}}{dt}$) with the derivative of the capacitor voltage ($\frac{dv_{c}}{dt}$) affects the current of charge flowing through the output inductor, $L_{g}$[38]; therefore, the dynamics of the inductor current $L_{g}$ are taken into account, to ensure a precise power supply from the distributed generator.

The CF from Equation (5) is included in the controller, which will be explained later in this manuscript, and is shown in the Appendix A.1.

To fix the switching frequency to be obtained as the output of the VSI, a modulator stage is included, using an SVM [34].

For each sector of the SVM, $S_{j}$, of the valid switching states of the two-level, three-phase VSI, it is mandatory to obtain the prediction of the capacitor voltage vectors for the next sampling time, using the discrete-time model of the LCL filter. Then, these predictions are independently evaluated in the CF of Equation (5) [34].

Subsequently, the following set of equations has to be solved, to obtain the duty cycles for each sector of the SVM [39]:

(6) $$\begin{array}{c} d_{0}=K/g_{0}\\ d_{1}=K/g_{1}\\ d_{2}=K/g_{2}\\ d_{0}+d_{1}+d_{2}=T_{s}, \end{array}$$

where $d_{0}$ is the duty cycle of a zero vector that is evaluated only once.

To find the value of *K*, the set of equations of (6) has to be worked out, providing, as a result, the duty cycles for each vector:

(7) $$\begin{array}{c} d_{0}=\frac{T_{s}\cdot g_{1}\cdot g_{2}}{\left(g_{0}\cdot g_{1}+g_{1}\cdot g_{2}+g_{0}\cdot g_{2}\right)}\\ d_{1}=\frac{T_{s}\cdot g_{0}\cdot g_{2}}{\left(g_{0}\cdot g_{1}+g_{1}\cdot g_{2}+g_{0}\cdot g_{2}\right)}\\ d_{2}=\frac{T_{s}\cdot g_{0}\cdot g_{1}}{\left(g_{0}\cdot g_{1}+g_{1}\cdot g_{2}+g_{0}\cdot g_{2}\right)}. \end{array}$$

From the equations in (7), the new cost function, which is evaluated at every sampling time, $T_{s}$, is defined as:

(8) $$g\left(k+1\right)=d_{1}\cdot g_{1}+d_{2}\cdot g_{2}.$$

A new CF is defined in Equation (8), in which the obtained vectors which minimise it are applied to the VSI at the next sampling period. When the duty cycles have been obtained, and the optimal vectors to be applied have been chosen, the switching pattern is utilised, for the two active vectors and the two zero vectors to be used [34,39].

This implementation is configured in the dSPACE ds1103 control platform, using the concept shown in Figure 2. To create the SVM with the dSPACE ds1103, its slave DSP, that is I/O available, is used.

**Figure 10:**
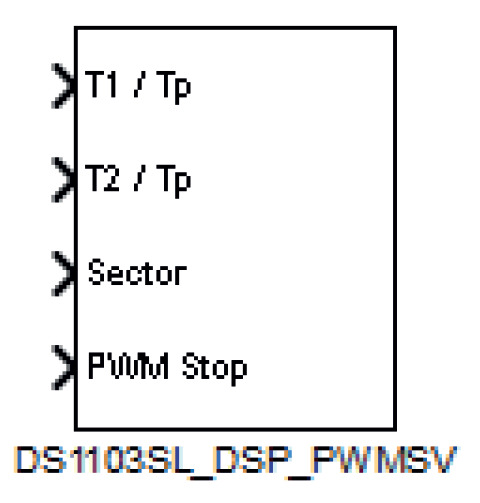
Block DS1103SL_DSP_PWMSV, available in the slave DSP contained in the dSPACE ds1103 control platform.

Embedded in the MATLAB/Simulink environment, the dSPACE’s Simulink library contains a block called ’DS1103SL_DSP_PWMSV’, which generates the three-phase space vector PWM with original and inverted outputs and, if needed, a variable deadband (Figure 10) [28].

The Simulink block DS1103SL_DSP_PWMSV, shown in Figure 10, generates space vector PWM signals that are used to implement the SVM in the controller. Thus, the parameters from the M${}^{2}$PC scheme are $t_{1}$ and $t_{2}$, as follows [28]:

(9) $$t_{1}+t_{2}\leq t_{p},$$

with $t_{p}$ as the value of the period.

These parameters from the modulated model predictive controller embedded in the ‘S-Function Builder’ block are sent to the DS1103SL_DSP_PWMSV block shown in Figure 10.

The space vector determines the sector and the values $t_{1}$ and $t_{2}$ of the corresponding right ($t_{1}$) and left ($t_{2}$) vectors. The expression $t_{1}/t_{p}$ denotes the duty cycle of the right vector in the corresponding sector, while $t_{2}/t_{p}$ denotes the duty cycle of the left vector. The sector, which is in the range from 1 to 6, is defined by reflecting the space vector onto the plane determined by the basic space vectors. The values $t_{1}$ and $t_{2}$ are defined by the projection of the space vector onto the two adjacent basic space vectors [37].

**Figure 11:**
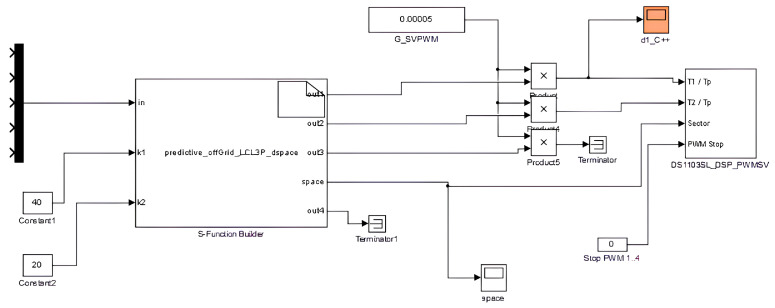
View of the output of the controller, with $t_{1}$ and $t_{2}$ entering into the DS1103SL_DSP_PWMSV block in the dSPACE’s Simulink environment.

To make the fixed switching frequency work at the desired frequency, in Figure 11, it can be seen that there is a multiplier for the controller outputs, fixing the switching frequency at 20 kHz (0.00005) (see Table 2).

In Figure 11, it can be seen that the weighting factors $\lambda_{i_{o}}$ and $\lambda_{v_{f}}$—referred to as $k_{1}$ and $k_{2}$, respectively, in the Simulink model—included in the CF of Equation (5), are introduced to the controller in the ‘S-Function Builder’ block.

The modulated model predictive controller is embedded into the ’S-Function Builder’ block, which can be seen in Figure 11. Inside this block, the S-function can be built to operate as the controller for the Simulink model for the fixed-switching-frequency M${}^{2}$PC.

The complete code of the fixed-switching-frequency modulated model predictive controller is included in Appendix A.1.

#### 3.2.2. Droop Control and Virtual Impedance

To establish the power sharing among the two VSIs of the RL load, the opposite droop equations are considered, as detailed in [34]:

(10) $$E_{ref}=E_{nom}-k_{p}\;P_{cal};$$

(11) $$\omega_{ref}=\omega_{nom}+k_{q}\;Q_{cal},$$

where $E_{ref}$ and $\omega_{ref}$ are the reference voltage amplitude and frequency used to obtain $v_{ref}$, which is introduced to the virtual impedance loop in the following equation, as a virtual resistive loop, $R_{v}$  [34]:

(12) $$v_{f}^{*}=v_{ref}-R_{v}\cdot i_{oabc},$$

and with $E_{nom}$ and $\omega_{nom}$ as the nominal voltage amplitude and frequency, respectively.

To calculate $P_{cal}$ and $Q_{cal}$, the following equations are used [34]:

(13) $$P_{cal}=v_{f\alpha }\;i_{o\alpha }+v_{f\beta }\;i_{o\beta };$$

(14) $$Q_{cal}=v_{f\beta }\;i_{o\alpha }-v_{f\alpha }\;i_{o\beta }$$

**Figure 12:**
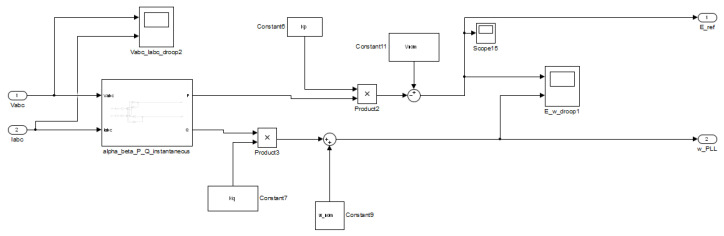
View of the droop control established in the dSPACE ds1103 platform’s Simulink environment.

**Figure 13:**
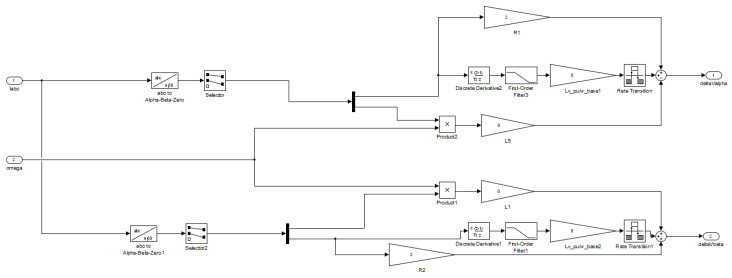
View of the resistive virtual impedance established in the dSPACE ds1103 platform’s Simulink environment.

The equations referring to the droop control and the implementation of the virtual impedance loop, (10)–(12), are implemented in the Simulink model for the dSPACE ds1103 platform, as shown in Figure 12 and Figure 13.

The computing of the instantaneous active and reactive power shown in Equations (13) and (14) are implemented in the Simulink model for the dSPACE ds1103 platform.

#### 3.2.3. Analogue-to-Digital Converters

**Figure 14:**
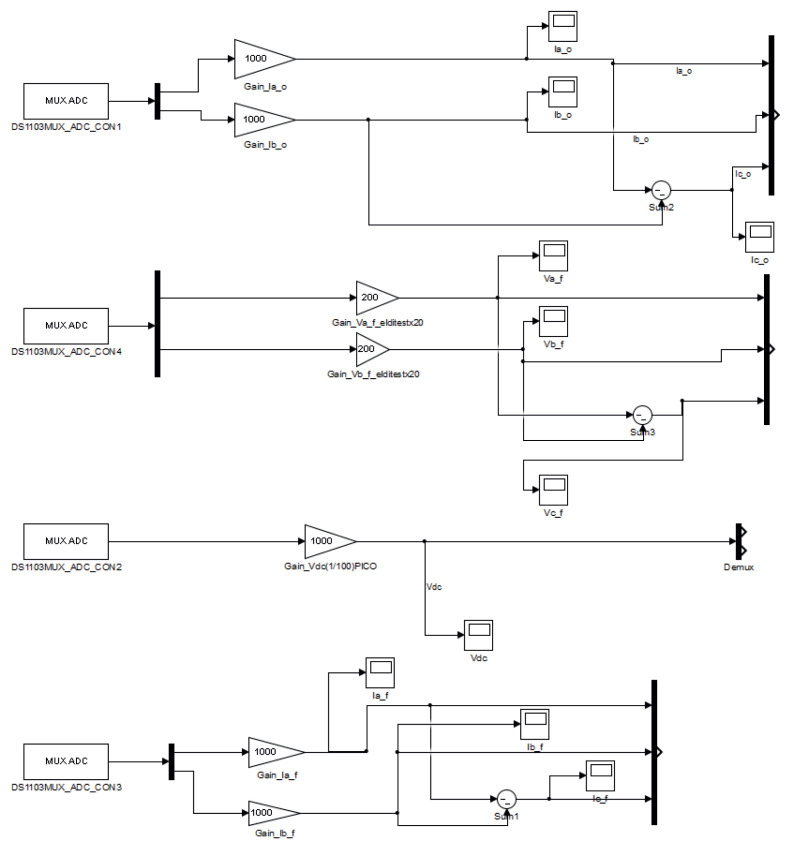
View of the ADCs dSPACE’s Simulink blocks to read the voltages and currents coming from the experimental setup.

To get VSI readings on the Simulink dSPACE ds1103 platform, the available ADCs were used. Current clamps and differential voltage probes were connected to the dSPACE ds1103 platform through these ADCs. As explained previously, the dSPACE platform had multiplexed and non-multiplexed ADCs. In Figure 14, on the left side, the ADC Simulink blocks can be seen. The name of the block corresponds to ‘DS1103MUX_ADC_CONX’ where ‘CONX’ refers to the number of the analogue-to-digital converter.

In addition, only multiplexed ADCs were used to obtain currents and voltages readings. Taking into account Figure 14, the ADCs used to obtain these readings were as follows:

- ADCH1 and ADCH2, for $i_{fa}$ and $i_{fb}$, respectively;
- ADCH3 and ADCH4, for $i_{oa}$ and $i_{ob}$, respectively;
- ADCH13 and ADCH14, for $v_{fab}$ and $v_{fbc}$, respectively;
- ADCH15 for $v_{dc}$.

Looking closely at Figure 14, it can be observed that only two phases of the filter current, $i_{fabc}$, of the output current, $i_{oabc}$, and of the capacitor voltage, $v_{fabc}$, were measured. Thus, the reading of the missing phase was obtained by subtracting the measurements of the other two phases.

#### 3.2.4. Interruption from the Slave DSP

In the slave DSP of the dSPACE ds1103, an interruption is available almost over the entire PWM period. The PWM interrupt (from slave to master) is triggered by the falling edge of the active-low synchronisation interrupt signal [37].

**Figure 15:**
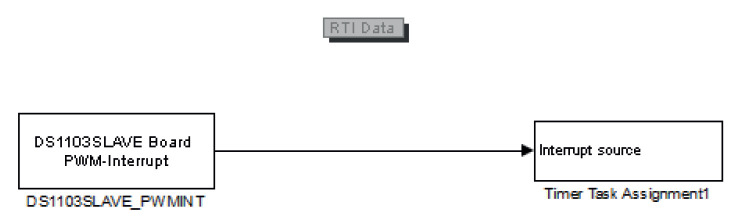
View of the dSPACE’s Simulink block DS1103SLAVE_PWMINT connected to the block ’Timer Task Assignment’ to make the slave DSP’s interruption available.

To make this slave DSP’s interruption available to the real-time simulation in the dSPACE ds1103’s Simulink, the Simulink block ’DS1103SLAVE_PWMINT’ has to be added as can be seen in Figure 15.

Alternatively, the interruption can come from an external source, and be available for real-time simulation.

In this work, the interruption came from the slave DSP of the dSPACE ds1103 platform, as shown in the general scheme from Figure 21.

#### 3.2.5. Frequency Reference

Considering that this microgrid is decoupled from the main grid, it is not necessary to use a phase-locked loop (PLL) strategy: that is, the operating frequency can be arbitrarily determined.

**Figure 16:**
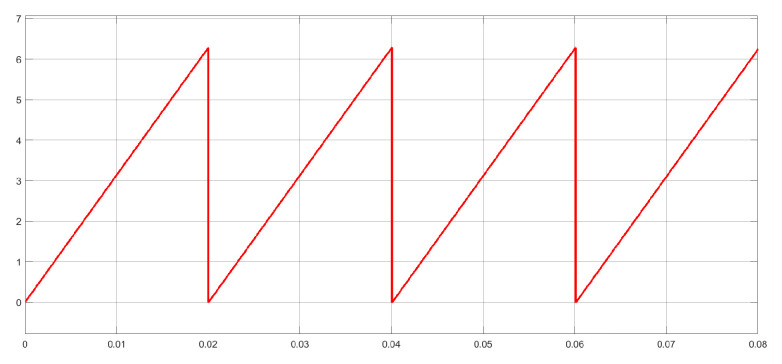
View of the $\theta_{ref}\left(k\right)$ being restarted when reaching 2π value.

For the implementation of the system, 50 Hz is set as the operating frequency; therefore, the argument of currents and voltages—that is, the operating angle $\theta_{ref}$—appears when integrating the angular speed according to what is shown in Equation (15). To generate $\theta_{ref}$ in the dSPACE ds1103,  Equation (16) has to be considered. In Equation (16), $\theta_{ref}\left(k\right)$ tends to infinity; therefore $\theta_{ref}\left(k\right)$ has to be restarted (taking 0 as the value) each time it reaches the value of 2π. This is shown in Figure 16.

(15) $$\begin{array}{c} \theta =\frac{T_{s}\omega_{ref}\left(z+1\right)}{2(z-1)} \end{array}$$

(16) $$\begin{array}{c} \theta_{ref}\left(k\right)=\theta_{ref}\left(k-1\right)+T_{s}\omega_{ref} \end{array}$$

### 3.3. FPGA

To complement the dSPACE ds1003 control platform, and connected via its I/O bus, an Atlys FPGA is included. This corresponds to a control board based on an Xilinx Spartan-6 FPGA (Figure 21).

The internal clock of the Atlys FPGA corresponds to the 100 MHz CMOS oscillator [40].

Additionally, the Atlys FPGA includes a Pmod port expansion, which allows the connection of the Digilent Vmod module interface board to interface additional peripheral modules to the Atlys FPGA [40,41].

**Figure 17:**
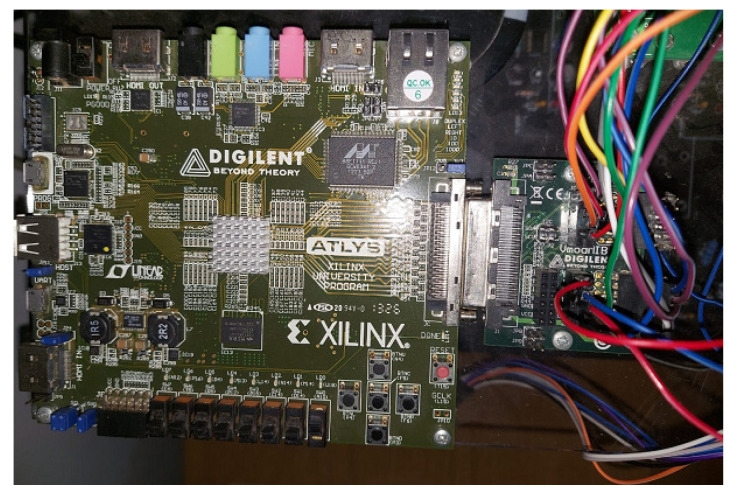
View of the Atlys FPGA with the Vmod module (VmodMIB) used in this work.

The VmodMIB shown in Figure 17 includes a VHDCI peripheral board connector and four HDMI and five 12-pin Pmod connectors [41]. As shown in Figure 21, several digital connections—the signals from the space vector PWM from the slave DSP of the dSPACE ds1103 control platform—are conducted through these expansion modules in the Atlys FPGA, to be processed, and the dead-times added for safe commutation of the VSI.

In this FPGA platform, the safe commutation of the VSI was implemented, to protect the two-level, three-phase VSI at the instant of switching. Thus, dead-times were programmed into the FPGA system. The latter was due to the fact that the codification of $S_{1}$, $S_{2}$, $S_{3}$, $S_{4}$, $S_{5}$, and $S_{6}$ into the new signals $S_{a}$, $S_{b}$, and $S_{c}$, did not guarantee, by itself, avoiding short-circuit in any of the power converter legs.

**Figure 18:**
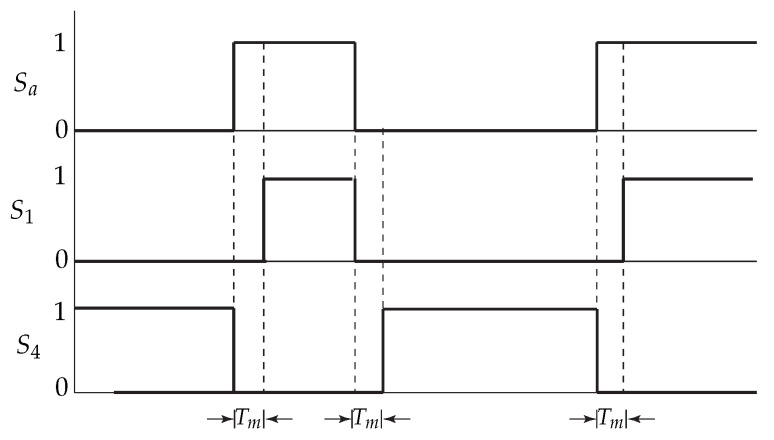
Dead-times applied to the commutation of the VSI’s first leg.

The concept of ’dead-time’ consists in opening both switches of one power converter’s leg at the moment that a change in the value of the signal $S_{a}$, $S_{b}$, or $S_{c}$ occurs. This opening of the switches is generated during a time instant ‘$T_{m}$’. The application of the dead-time to the commutation of the switches $S_{1}$ and $S_{4}$ can be seen in Figure 18.

Then, the dead-time, $T_{m}$, added to the safe commutation of the VSI corresponds to 1 μs (microseconds).

In Appendix A.2, a code subsection is shown for the addition of dead-time in the Atlys FPGA—in this case, for the first leg of the two-level, three-phase VSI.

### 3.4. dSPACE Control Desk

Once the Simulink model is developed, with everything required to establish the fixed-switching-frequency M${}^{2}$PC, the Simulink model has to be compiled, to build a ‘.sdf’ file, in order to implement the model into the dSPACE’s ControlDesk control software.

**Figure 19:**
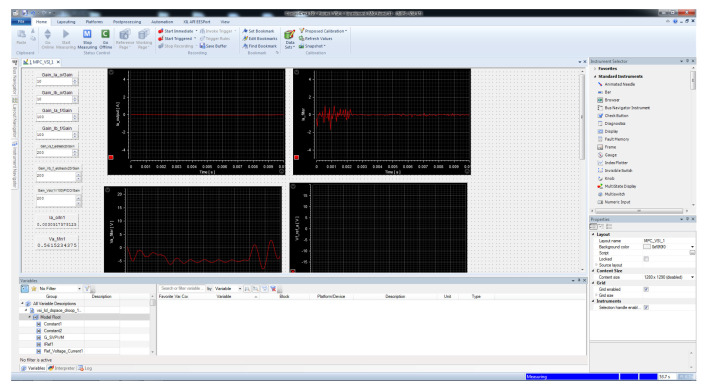
View of the dSPACE’s ControlDesk software platform to monitor the real-time simulation of the M${}^{2}$PC of the two-level, three-phase VSI.

In this ControlDesk software (Figure 19), it is possible to adjust the gains for the ADCs coming from the current clamps and the differential voltage probes, to make proper readings and to control the two-level, three-phase VSI.

From the ControlDesk software, the real-time simulation can be controlled, to go online, start measuring, and have graphical and numerical control of the relevant variables for the developed real-time simulation.

### 3.5. Complete Model

**Figure 20:**
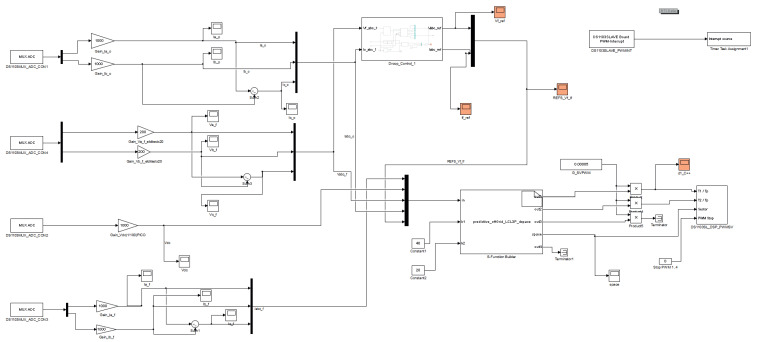
View of the dSPACE ds1103’s Simulink model for establishing the controller detailed in Figure 9.

In this subsection, the dSPACE’s Simulink model, which is built into the ‘.sdf’ file with all its components, is shown in Figure 20.

**Figure 21:**
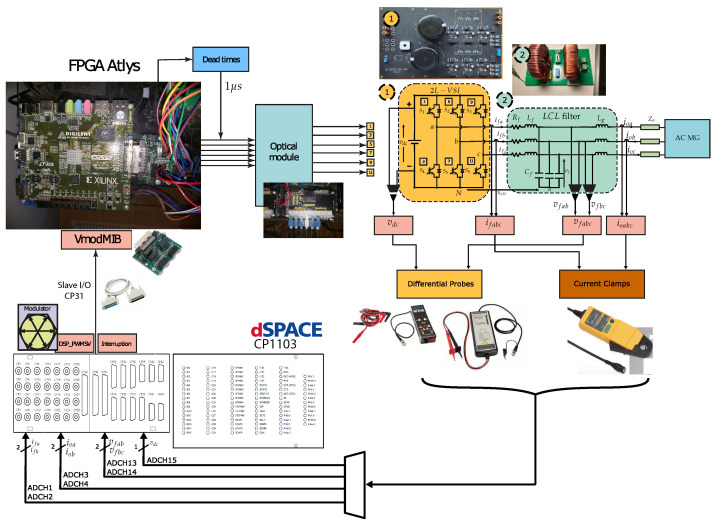
General concept of the establishment of the two-level, three-phase VSI setup controlled by a dSPACE ds1103 control platform plus an Atlys FPGA.

In the model shown in Figure 20, the interruption comes from the slave DSP that is contained in the dSPACE ds1130 control platform. Furthermore, the slave DSP generates the SVM using the Simulink block DS1103SL_DSP_PWMSV, as seen in Figure 20. These signals are sent to the Atlys FPGA, to add the dead-times for the final commutation signals to be sent through the optimal module, using optical fibre for each MOSFET switch of the two-level, three-phase VSI (Figure 21).

Filter currents, $i_{fabc}$, and output currents, $i_{oabc}$, are measured, using Fluke i30 and Fluke i310 current clamps. These current clamps are connected to the ADC panel of the dSPACE ds1103 (see Figure 21), and are interfaced into the Simulink model with the MUX ADC blocks, which can be seen in Figure 20. All gains are adjusted according to the resolution of every current clamp under use.

Similarly, the voltages of the system ($v_{fabc}$, and $v_{dc}$) are measured using the differential voltage probes, Elditest GE8115 and PICO TA043. The voltage measurements are introduced through the available ADCs in the control panel of the dSPACE ds11003. As in the current case, these ADCs are interfaced with the Simulink model by the ’MUX ADC blocks’, which can be seen in Figure 20. All gains are adjusted according to the resolution of every differential probe in use (see Figure 20).

However, every ADC entering through the correspondent ‘MUX ADC’ block into the Simulink model, requires a gain of an additional factor x10, that must be multiplied by every current clamp or differential voltage probe that obtains the current and voltage measurements, respectively.

As mentioned previously, for the measurements of the filter and output current, plus the capacitor voltages, only two phases are measured, and the *c* phase is calculated by subtracting the other two; the latter considering a balanced system. This can be clearly seen in Figure 20 and Figure 21.

## 4. Experimental Waveforms

In this section, the experimental results are included. The complete theoretical explanation of this work is developed extensively in a previous published work by the same authors, in [34].

Here, only the experimental results are included, as they were the result of the real-time simulation or hardware-in-the-loop, but using the real system of the LCL-filtered two-level, three-phase voltage source inverter. The complete setup was previously shown in Figure 8. Its parameters, and those used to implement the control, are shown in Table 2.

**Table 2:** Experimental parameters used in [34].

Parameter	Value
DC link voltage, $v_{dc}$	30 V
Switching frequency (M${}^{2}$PC)	20 kHz
Sampling time, $T_{s}$	50 μs
CF Weighting factors	$\lambda_{i_{o}}$ = 40, $\lambda_{v_{f}}$ = 20
LCL filter	$L_{f}$ = 2.0 mH, $C_{f}$ = 11 μF, $L_{g}$ = 1.0 mH
Load RL	$R_{l}$ = 10 Ω, $L_{l}$ = 10 mH
Nominal voltage	$E_{nom}$ = 15 V, $\omega_{nom}$ = 2π·50 Hz
Droop coefficients	$k_{p}$ = 0.0015 V/W, $k_{q}$ = 0.0025 rad/sVar
Line impedance	$R_{oi}$ = 0.1 Ω, $L_{oi}$ = 1.114 mH
Virtual resistance	$R_{v}$= 2 Ω

**Figure 22:**
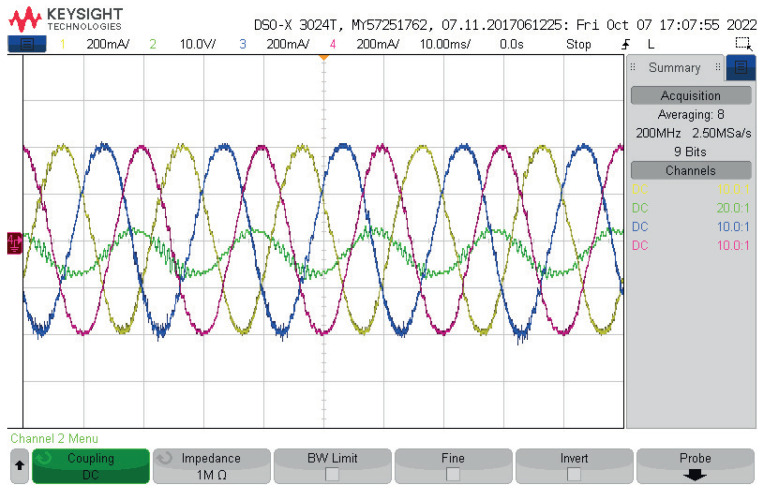
Experimental waveforms appeared in [34]: oscilloscope vista of the output currents, $i_{oa}$, $i_{ob}$, $i_{oc}$, and capacitor voltage, $v_{fa}$ of the two-level, three-phase VSI in the AC microgrid.

The waveforms obtained are shown in the oscilloscope-drawn view of Figure 22. These output currents were obtained after the LCL filter, and their shape looks perfectly sinusoidal, showing that proper results were obtained. Additionally, in Figure 22, the capacitor voltage of the phase *a*, $v_{fa}$ is shown, with similar features.

**Figure 23:**
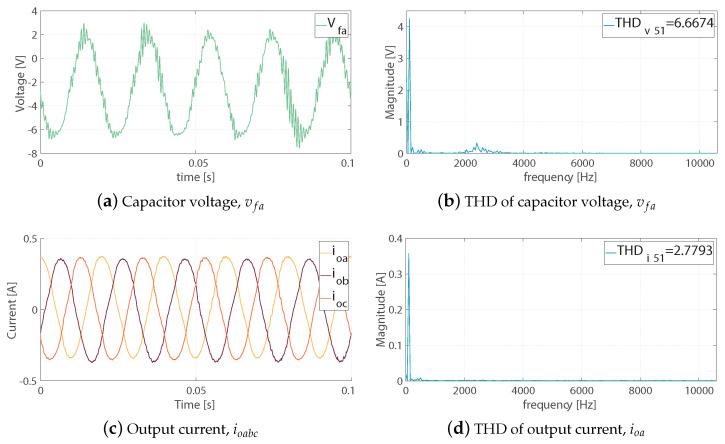
Experimental waveforms and THD for the two-level, three-phase VSI appeared in [34]: (**a**) capacitor voltage, $v_{fa}$; (**b**) THD of capacitor voltage, phase *a*, $v_{fa}$; (**c**) output current, $i_{oabc}$; (**d**) THD of output current, phase *a*, $i_{oa}$.

In Figure 23, the waveforms for the capacitor voltage phase *a* and the output currents can be seen. Additionally, their respective THD analyses are included.

## 5. Discussion

### 5.1. Experimental Waveforms

The experimental waveforms obtained using the real-time interface of the dSPACE ds1103 control platform are shown in Figure 22 and Figure 23. Although the experimental waveforms obtained were analysed in the previous published work in [34], some necessary discussion is included here.

Referring to the waveforms for the capacitor voltage of the phase *a*, $v_{fa}$:

- The capacitor voltage for phase *a*, $v_{fa}$, in Figure 22 and Figure 23a, was controlled as expected, but the presence of resonant noise can be seen. Resonance is known to be an inherent problem with LCL filters. As mentioned earlier in this article, the use of LCL filters was based on the availability of equipment in the laboratory; therefore, from these designs, the size of the capacitor had to be increased, from 1 μF to 11 μF, because, experimentally, an excess of resonance appeared;
- This resonance was produced by the parasitic components in the filters and in the semiconductor components (MOSFETs) of the experimental setup. Additionally, the fact that, as a contributor to this resonance in the capacitor voltage waveform, the delay provided by the drivers and the elements of the trigger pulse appeared;
- As shown in Figure 23b, the THD value was ≈ 6.6%, and was within the established standard deviation limits of 519–2014;
- The THD of the capacitor voltage, $v_{fa}$, the harmonics spectrum, was not spread across the frequencies, as mentioned earlier as the main drawback of not fixing the frequency.

Referring to the waveforms for the output currents, $i_{oabc}$:

- The waveforms of the output currents, $i_{oabc}$, are shown in Figure 23c;
- Spectra analysis is shown in Figure 23d, in which it can be seen that the low-frequency harmonics in the currents (500 Hz) may have been occasioned by the unbalance among the LCL filters and the RL load;
- The harmonics content was in the proximity of the limits of the 51st harmonics, with the currents having less distortion (≈2.8%), with attenuated high frequencies, by the filter inductances and the microgrid system.

Finally, the obtained experimental waveforms clearly show that application of the fixed-switching-frequency M${}^{2}$PC scheme controlled the LCL-filtered two-level, three-phase VSI, obtaining proper waveforms for the capacitor voltages and the output currents, while allowing a balance in the whole microgrid system.

### 5.2. dSPACE Implementation

In this work, the implementation of a modulated model predictive control of a two-level, three-phase VSI in an islanded AC microgrid using a dSPACE ds1103 control platform was explained, step-by-step, in detail.

The two-level, three-phase VSI was developed in the laboratory, with a PCB especially designed for that purpose. The switches used were described, to facilitate understanding of their features, which may affect the implementation of the control of the power converters embedded in the experimental islanded AC microgrid.

In the outputs of each phase of the VSI, LCL filters were included, to ease the filtering of the harmonics of the obtained currents, and to project an eventual future experimental development for on-grid AC microgrids. The experimental characteristics of the LCL filters were described, as well as those of the PCB bases for the construction of the LCL filter developed in the laboratory. The mathematical discrete model was briefly described, to give context to the implementation of the modulated model predictive controller for the real-time simulation in the dSPACE ds1103 control platform. The RL load was characterised by providing its parameters, and pinpointing its location in the experimental setup. In this way, it was explained that the setup considered this shared load by the two paralleled-VSIs.

In order to better explain the implementation of this dSPACE-based AC microgrid, the technical features of the dSPACE ds1103 control platform were described, providing details of the internal microcontrollers (slave DSP), the connectors that were used for the implementation of the M${}^{2}$PC, and the ADCs that were used for obtaining the measurements of the filter and output currents, the capacitor voltages, and the DC-link voltage. A very important aspect of the dSPACE ds1103 control platform, which was essential to implementing the M${}^{2}$PC, was the possibility of using the space vector PWM signals generated by the internal slave DSP microcontroller. These signals were then introduced into the Atlys FPGA platform for final processing, by adding dead-times for safe commutation of the power converter.

The implementation of the controller was deployed in the real-time interface of the dSPACE ds1103 control platform, which allowed the use of MATLAB/Simulink with real equipment, the experimental setup of the islanded AC microgrid. A brief explanation of the fundamentals of the M${}^{2}$PC was provided, considering the cost function to be minimised, where the control objectives were defined (in this case, output currents and capacitor voltages). Then, the modulator was briefly explained, also, showing that using the SVM and its active vectors that form every SVM sector was evaluated in the cost function for every sampling time. Then, the calculation of the duty cycles for each sector of the SVM was explained, and then evaluated in the new cost function. At this point, it was explained how the SVM was implemented in the dSPACE-based AC microgrid, using space vector PWM from the internal slave DSP of the dSPACE ds1103. In addition, it was explained where to obtain these signals for the controller implementation, by using the available library for the ds1103 slave DSP in Simulink. Then, the inputs of the SVM were explained, as they were the outputs of the model-predictive stage. The complete Simulink for the M${}^{2}$PC was shown, paying attention to the relevant blocks that allowed for interaction between the real equipment and the software-simulated environment.

Continuing with the explanation of the controller implementation, the code for the M${}^{2}$PC, and an illustration of how this code was embedded in the Simulink model, as the S-Function embedded in the Simulink model, were fully included in Appendix A.1 of this paper.

Referring to power sharing control, the droop control and virtual impedance were briefly explained, and their modelling in the Simulink model were shown and explained.

The use of the available ADCs was explained, describing how to include them in the Simulink model, and how their distribution was used to obtain the filter and output currents, the capacitor voltages, and the DC voltage.

The slave DSP interruption used in this real-time simulation was explained, showing the Simulink block that allowed it to be available for the implementation of the real-time simulation.

The method used to determine the frequency reference in the Simulink model was explained, showing that, as the AC microgrid operated off-grid, a phase-locked loop algorithm was not needed.

A crucial part of the implementation was the determination of the dead-times to be included in the commutation signals that went to the real plant, the two-level, three-phase VSI. This was the process that was carried out in the FPGA, and the details of its programming are given in detail in Appendix A.2, where the code for the addition of dead-times for the first leg of the VSI is shown.

The dSPACE developers included a complete software platform for testing and monitoring dSPACE real-time systems simulations. This ControlDesk software uses a file that is compiled from the Simulink model described at the outset. This software platform allowed the complete control and monitoring of the relevant variables for this implementation. Gains for the several ADCs used were able to be adjusted, to find proper measurements of the filter and output currents, and for the capacitor voltages and DC voltage. Additionally, the graphical environment allowed for proper visualisation of the functioning of the control for the input and output variables relevant to this implementation.

The obtained experimental waveforms were included in the paper, to show that the experimental implementation of the modulated model predictive control for the two-level, three-phase voltage source inverter in a dSPACE-based AC microgrid is completely feasible, including power sharing control with the droop control loop and with the added virtual resistive impedance.

## 6. Conclusions

This article seeks to contribute relevant information on the process of implementing an experimental islanded AC microgrid with two-level, three-phase voltage source inverters, LCL filters, and sharing an RL load, using a real-time simulation as the modulated model predictive controller. The implementation of the controller was realised on the real-time interface available for the dSPACE ds1103 control platform and its internal slave DSP microcontroller.

The fixed-switching frequency had a value of 20 kHz; a value that did not present any problem for the dSPACE ds1103 control platform, and did not cause any overrun problem to it.

The MATLAB/Simulink working environment was quite simple to use, and the inclusion of the RTI dSPACE libraries was essential to implementing the modulated model predictive controller.

There is a lack of fully documented hardware-in-the-loop implementations and real-time simulations of power converters, microgrids, and distributed generation.

Real-time simulation and hardware-in-the-loop control platforms will continue to play a very relevant role in prototyping and testing in several engineering fields. In particular, for power converters, microgrids and distributed generation will continue to allow researchers to implement and test several systems architectures, and several advanced control strategies.

Finally, highlighting the contribution of this article, a summary, Table 3, is presented below, which compares all articles surveyed on real-time and hardware-in-the-loop control platforms, considering whether or not they include a detailed step-by-step explanation of the implementation of the control strategy developed for each article.

## Figures and Tables

**Table 1:** MOSFET STF22N60M6 electrical ratings [35].

Parameter	Value
Gate–source voltage, $V_{GS}$	±25 V
Drain current (continuous) at $T_{case}=25\;{}^{\circ }C$, $I_{D}$	15 A
Drain current (continuous) at $T_{case}=100\;{}^{\circ }C$, $I_{D}$	9.5 A
Drain current (pulsed), $I_{DM}$	42 A
Total power dissipation at $T_{case}=25\;{}^{\circ }C$, $P_{TOT}$	30 W
Peak diode recovery voltage slope, dv/dt	15 V/ns
MOSFET dv/dt ruggedness, dv/dt	100 V/ns
Insulation withstand voltage (RMS)	
(t=1 s; $T_{case}=25\;{}^{\circ }C$), $V_{ISO}$	2.5 kV
Storage temperature range, $T_{stg}$	−55 to $150\;{}^{\circ }C$
Operating junction temperature range, $T_{j}$	−55 to $150\;{}^{\circ }C$

**Table 3:** Summary of works, including hardware-in-the-loop (HIL) and real-time simulation platforms.

Ref./Year	Control Strategy	Control Platform	Contribution to HIL Use
[21]/2018	FS-MPC (M${}^{2}$PC)	dSPACE ds1103	Detailed step-by-step implementation not included.
[29]/2018	FS-MPC	dSPACE MicroLabBox ds1202	Detailed step-by-step implementation not included.
[30]/2013	Linear, PI control	dSPACE ds1103	The use of Simulink blocks explained, but not giving further details of the Simulink model.
[31]/2021	FS-MPC (M${}^{3}$PC)	dSPACE ds1104	Detailed step-by-step implementation not included.
[32]/2018	FS-MPC	Opal OP5700 RT-LAB	Detailed step-by-step implementation not included.
[33]/2021	FS-MPC (M${}^{2}$PC)	Typhoon HIL 402.	Detailed step-by-step implementation not included.
[25]/2010	Linear control for DESS	Opal RT-LAB	Construction process in MATLAB/Simulink is described, but more details are needed.
[27]/2020	Lyapunov function.	dSPACE 1104	Inclusion of the complete control system as Simulink model for the dSPACE ds1104 platform.
This paper	FS-MPC (M${}^{2}$PC)	dSPACE ds1103	The step-by-step implementation process explained for the Simulink model for the dSPACE ds1103 platform.

## Abbreviations

    The following abbreviations were used in this manuscript:

AC	alternating current
PCC	point of common coupling
M${}^{2}$PC	modulated model predictive control
VSI	voltage source inverter
MPC	model predictive control
FS-MPC	finite-set model predictive control
DG	distributed generator
DER	distributed energy resource
MG	microgrid
HIL	hardware-in-the-loop
PID	proportional–integral–differential
PI	proportional–integral
RT-LAB	real-time laboratory
M${}^{3}$PC	modified modulated model predictive control
DFIG	doubly fed induction generator
RTI	real-time interface
SVM	space vector modulation
PWMSV	pulse-width modulation space vector
PWMINT	pulse-width modulation interruption
HWINT	hardware (external) interruption
CF	cost function
NPC	neutral-point-clamped
LCL	inductor–capacitor–inductor
MOSFET	metal-oxide-semiconductor field-effect transistor
PWM	pulse-width modulation
DC	direct current
RL	resistor–inductor
DSP	digital signal processor
THD	total harmonic distortion
FPGA	field-programmable gate array
I/O	input–output
ADC	analogue-to-digital-converter
DAC	digital-to-analogue-converter
LED	light-emitting diode
PLL	phase-locked loop
PCB	printed circuit board
RMS	root mean square
LERAE	laboratory of renewable energy and electrical conditioning
DESS	distributed energy storage system

## Appendix A. Code of the Modulated Predictive Controller and the Dead-Times Generation with the Atlys FPGA

### Appendix A.1. Code of the Modulated Model Predictive Control

        
/*Measured inverter currents alpha beta/*

iaINV=in[0];

ibINV=in[1];

icINV=in[2];

        
/*Measured inverter currents/*

i_alphaINV=(2*iaINV-ibINV-icINV)/3;

i_betaINV=(ibINV-icINV)/sqrt(3);

        
/*DC link voltage/*

Vdc=in[3];

        
/*Capacitor voltages/*

Vac=in[4];

Vbc=in[5];

Vcc=in[6];

        
/*Alpha beta capacitor voltages/*

Vc_alpha=(2*Vac-Vbc-Vcc)/3;

Vc_beta=(Vbc-Vcc)/sqrt(3);

        
/*Measured load currents/*

iaLoad=in[7];

ibLoad=in[8];

icLoad=in[9];

        
/*Measured load currents alpha beta/*

i_alphaLoad=(2*iaLoad-ibLoad-icLoad)/3;

i_betaLoad=(ibLoad-icLoad)/sqrt(3);

        
/*REFERENCES/*

/*Reference capacitor voltages/*

Vac_ref=in[10];

Vbc_ref=in[11];

Vcc_ref=in[12];

        
/*REF alpha beta capacitor voltages/*

VcREF_alpha=(2*Vac_ref-Vbc_ref-Vcc_ref)/3;

VcREF_beta=(Vbc_ref-Vcc_ref)/sqrt(3);

        
/*Reference inverter currents/*

iaINV_ref=in[13];

ibINV_ref=in[14];

icINV_ref=in[15];

        
/*Measured inverter currents/*

iREF_alphaINV=(2*iaINV_ref-ibINV_ref-icINV_ref)/3;

iREF_betaINV=(ibINV_ref-icINV_ref)/sqrt(3);

        
/*PARAMETERS/*

Ts=0.00005;

L1=2e-3;

L2=1e-3;

C=11e-6;

gopt=1e10;

        
/*Weight factors*/

k1x=k1[0];

k2x=k2[0];

        
ialphaINV_0 = i_alphaINV + (Ts/L1)*(-Vc_alpha);

ibetaINV_0 = i_betaINV + (Ts/L1)*(-Vc_beta);

Vc_alpha0 =Vc_alpha + (Ts/C)*(ialphaINV_0-i_alphaLoad);

Vc_beta0 =Vc_beta + (Ts/C)*(ibetaINV_0-i_betaLoad);

        
g_0alpha = k1x*k1x*(iREF_alphaINV - ialphaINV_0)*(iREF_alphaINV -

ialphaINV_0)+k2x*k2x*(VcREF_alpha - Vc_alpha0)*(VcREF_alpha - Vc_alpha0);

g_0beta = k1x*k1x*(iREF_betaINV - ibetaINV_0)*

(iREF_betaINV - ibetaINV_0)+k2x*k2x*(VcREF_beta - Vc_beta0)*

(VcREF_beta - Vc_beta0);

g_0=g_0alpha+g_0beta;

        
/*PREDICTIONS/*

        
for(k=0;k<6;k++)

{

VaN=(vectores[vec[k][0]][0]-0.5)*Vdc;

VbN=(vectores[vec[k][0]][1]-0.5)*Vdc;

VcN=(vectores[vec[k][0]][2]-0.5)*Vdc;

Vx= -(VaN+VbN+VcN)/3;

VaN=VaN+Vx;

VbN=VbN+Vx;

VcN=VcN+Vx;

        
ValphaN=(2*VaN-VbN-VcN)/3;

VbetaN=(VbN-VcN)/sqrt(3);

        
ialphaINV_1 = i_alphaINV + (Ts/L1)*(ValphaN-Vc_alpha);

ibetaINV_1 = i_betaINV + (Ts/L1)*(VbetaN-Vc_beta);

        
Vc_alpha1 =Vc_alpha + (Ts/C)*(ialphaINV_1-i_alphaLoad);

Vc_beta1 =Vc_beta + (Ts/C)*(ibetaINV_1-i_betaLoad);

        
g_1alpha = k1x*k1x*(iREF_alphaINV - ialphaINV_1)*

(iREF_alphaINV - ialphaINV_1)+k2x*k2x*(VcREF_alpha - Vc_alpha1)*

(VcREF_alpha - Vc_alpha1);

g_1beta = k1x*k1x*(iREF_betaINV - ibetaINV_1)*(iREF_betaINV - ibetaINV_1)+

k2x*k2x*(VcREF_beta - Vc_beta1)*(VcREF_beta - Vc_beta1);

g_1=g_1alpha+g_1beta;

        
/*Second Prediction/*

        
VaN=(vectores[vec[k][1]][0]-0.5)*Vdc;

VbN=(vectores[vec[k][1]][1]-0.5)*Vdc;

VcN=(vectores[vec[k][1]][2]-0.5)*Vdc;

Vx= -(VaN+VbN+VcN)/3;

VaN=VaN+Vx;

VbN=VbN+Vx;

VcN=VcN+Vx;

        
ValphaN=(2*VaN-VbN-VcN)/3;

VbetaN=(VbN-VcN)/sqrt(3);

        
ialphaINV_1 = i_alphaINV + (Ts/L1)*(ValphaN-Vc_alpha);

ibetaINV_1 = i_betaINV + (Ts/L1)*(VbetaN-Vc_beta);

        
Vc_alpha1 =Vc_alpha + (Ts/C)*(ialphaINV_1-i_alphaLoad);

Vc_beta1 =Vc_beta + (Ts/C)*(ibetaINV_1-i_betaLoad);

        
g_2alpha = k1x*k1x*(iREF_alphaINV - ialphaINV_1)*

(iREF_alphaINV - ialphaINV_1)+k2x*k2x*(VcREF_alpha - Vc_alpha1)*

(VcREF_alpha - Vc_alpha1);

g_2beta = k1x*k1x*(iREF_betaINV - ibetaINV_1)*

(iREF_betaINV - ibetaINV_1)+k2x*k2x*(VcREF_beta - Vc_beta1)*

(VcREF_beta - Vc_beta1);

g_2=g_2alpha+g_2beta;

        
d1 = ((g_0*g_2)/(g_1*g_2 + g_0*g_2 + g_0*g_1));

d2 = ((g_0*g_1)/(g_1*g_2 + g_0*g_2 + g_0*g_1));

d0 = ((g_2*g_1)/(g_1*g_2 + g_0*g_2 + g_0*g_1));

        
g = (d1*g_1 + d2*g_2);

        
if(g<gopt)

{

gopt = g;

T0 = d0*10000;

T1 = d1*10000;

T2 = d2*10000;

vec_1 = vec[k][0];

vec_2 = vec[k][1];

pos=k;

}

}

        
out1[0] = T1;

out2[0] = T2;

out3[0] = T0;

space[0] = pos+1;

### Appendix A.2. Code of Dead-Times in the Atlys FPGA for the First Leg of the Two-Level, Three-Phase VSI

        
‘define dead_time 101

        
reg [32:0] aux1;

reg [32:0] aux2;

begin

aux1=0;

initial

aux2=0;

end

        
always @(posedge mclk)

begin

        
begin

aux1=aux1+1;

if (JB0==1 && sw0==1)

aux2=0;

JA1=0;

end

        
begin

JA0=0;

if (JB0==0 && sw0==1)

aux1=0;

aux2=aux2+1;

end

        
begin

JA0=1;

if (aux1==‘dead_time && sw0==1)

end

        
begin

JA1=1;

if (aux2==‘dead_time && sw0==1)

end

        
if (sw0==0)

begin

JA0=0;

JA1=0;

end

        
end
